# Organic cultivation practices enhanced antioxidant activities and secondary metabolites in giant granadilla (*Passiflora quadrangularis* L.)

**DOI:** 10.1371/journal.pone.0255059

**Published:** 2021-07-26

**Authors:** Shiamala Devi Ramaiya, Huei Hong Lee, Yong Jun Xiao, Nur Shahirah Shahbani, Muta Harah Zakaria, Japar Sidik Bujang

**Affiliations:** 1 Department of Crop Science, Faculty of Agriculture Sciences and Forestry, Universiti Putra Malaysia Bintulu Campus, Bintulu, Sarawak, Malaysia; 2 International Food and Water Research Centre, Waters Pacific Pte Ltd, Singapore, Singapore; 3 Department of Aquaculture, Faculty of Agriculture, Universiti Putra Malaysia, Serdang, Selangor Darul Ehsan, Malaysia; 4 Department of Biology, Faculty of Science, Universiti Putra Malaysia, Serdang, Selangor Darul Ehsan, Malaysia; Bangabandhu Sheikh Mujibur Rahman Agricultural University, BANGLADESH

## Abstract

*Passiflora quadrangularis* L. belongs to the family Passifloraceae which bears larger fruit with edible juicy mesocarp and pulp known as a good source of phytochemicals. Cultivation and plant management practices are known to influence the phytochemical compositions of agricultural produce. This study aimed to examine the influence of the cultivation practices on the antioxidant activities and secondary metabolites of the organically and conventionally grown *P*. *quadrangularis*. Findings revealed organically treated *P*. *quadrangularis* plants showed enhancement in their antioxidant properties and secondary metabolites profiles. Among the plant parts, leaves of *P*. *quadrangularis* grown organically possessed higher antioxidant activities compared to the conventional in all assays evaluated. The antioxidant activities in the edible parts of the *P*. *quadrangularis* fruit have also been enhanced through organic cultivation with significantly higher total phenolic content and DPPH in mesocarp, and the pulp showed higher total flavonoid content, DPPH and FRAP. This observation is supported by a higher level of vitamins and secondary metabolites in the samples. The secondary metabolites profile showed mesocarps were phenolic rich, the pulps were flavonoids rich while leaves showed good composition of phenolics, flavonoids and terpenoids with outstanding antioxidant activities. The common secondary metabolites for organically produced *P*. *quadrangularis* in different plant parts include 2-isopropyl-3-methoxycinnamic acid (mesocarp and pulp), myricetin isomers (pulp and leaves), and malvidin-3-O-arabinoside isomers (pulp and leaves). This study confirmed that organic cultivated *P*. *quadrangularis* possessed higher antioxidant activities contributed by its vitamins and secondary metabolites.

## Introduction

The green revolution that fulfilled the growing population’s food demands caused an increase in yield per unit area in crop production. The use of chemical fertilizer has become widespread for addressing global food security challenges caused by low soil fertility. The use of chemical fertilizers has been important to replenish soil nutrients and enhance the quantity and quality of the agriculture produces. The intensive usage of chemical fertilizers to compensate for nutrient deficiency has resulted in soil degradation and environmental pollutions due to leaching by rain and heavy irrigation [1, 2]. According to Mozner et al. [3]. it has been found that crop only take up 30–50% of chemical fertilizers, and the remaining are lost to the environment.

Conversely, organic fertilizer has been reported to enhance the soil biological activities, chemical, and physical properties, thus increase plant growth and yield [4, 5]. The application of organic fertilizers sustains the cropping system through better nutrient recycling and has a significant beneficial effect on food production worldwide [6]. There is abundant anecdotal evidence indicating that organic manures have enhanced antioxidant activities and secondary metabolites, i.e., flavonoid, phenolic, lycopene and beta-carotene, contents than conventionally produced fruits and vegetables [7–9].

The presence of bioactive compounds in foods, mainly fruits, vegetables, and nuts, provide health benefits beyond the essential nutritional value and quality of their tastes, color, and organoleptic characteristics [10, 11]. The plant bioactive compounds-rich diets offer important protections against the development and progression of many chronic pathological conditions, including cardiovascular problems, diabetes, hypertension, cancer, and ageing [12, 13]. A higher level of phenolic content and stronger antioxidant activity was recorded for organically grown passion fruit [14]. Organic raspberry of ‘Kweli’ cultivar fruits contain significantly higher total phenols than the conventional practice [15]. The differences between secondary metabolites constituents in organically and conventionally produces allows for the possibility that organically grown plants may benefit human health better, and this has led to consumers’ increasing preference for organic produces [16, 17].

*Passiflora* species are known to be one of the most alluring and appealing plants of the tropics. The demand for passion fruit increases because of its organoleptic properties, its essential nutrient compositions, multi-vitamins contents, and antioxidant properties. Several species have a long history in traditional herbal therapy, but the medicinal use has been scientifically verified only for very few *Passiflora* species. In particular, *P*. *incarnata* L. is the most widely used top address anxiety disorders in contemporary Western phytotherapy [18].

In recent years, *P*. *quadrangularis*, also known as giant granadilla, gain attention due to its larger fruits size (~ 1–3 kg) with aromatic flavour, and also its health benefits. This species has been cultivated to a limited extent for local consumption at certain countries, including Malaysia. This fruit is known as ’badea’ in Colombia with the production more than 1000 tons of fruits per year [19], and its pulp is widely used for juice production while the mesocarp also edible. In folk medicine, leaves of this species are used as a sedative and mild tranquilizer. This finding is further supported by Gazola et al. [20]. where the flavonoid fractions and vitexin-2"-*O*-xyloside (V2OX) induced the sedative activity in mice. Additionally, Ingale and Hivrale [21] reported that this plant is used by traditional healers to treat the haemorrhaging effect caused by snake bites. These proposed it potential as active ingredient of herbal medicines and contributing to the value of *P*. *quadrangularis*.

From the above review, although some authors have reported the secondary metabolite present in the by-products (i.e., leaves) of *P*. *quadrangularis*, the antioxidant activity and secondary metabolites present in the pulp and mesocarp are less explored. Moreover, less research has been published on the impact of the cultivation practices on bioactive compounds made use of the metabolites profiling techniques for *P*. *quadrangularis*. This approach will provide better insight on the metabolite’s profiles in *Passiflora* genus. Therefore, the present study aimed to investigate the impact of the cultivation system, organic and conventional practices on the antioxidant activities and secondary metabolites of *P*. *quadrangularis* leaves and fruits. This study will help the researcher to uncover the potential uses of *P*. *quadrangularis* as a functional food and their importance for practical application in the nutraceutical and pharmaceutical industries.

## Materials and methods

### Study location

The present study was conducted in the passion fruit farm at Universiti Putra Malaysia Bintulu (N 03° 12.45’ and E 113° 4.68’), Sarawak from May 2019 to March 2020. The soil was categorized as Bekenu Series, Typic Paleudults) with sandy loam texture and the pH were 5.36–5.64 for depth of 0–15 cm. The climate variables, i.e., monthly rainfall, mean surface temperature, relative humidity, and sunshine hours for Bintulu, were obtained from the Malaysian Meteorological Department, Sarawak Branch (Kuching, Sarawak) daily from January 2019 to March 2020. During the study period, the average annual temperature was 26.6–27.8 ^o^C, while the average rainfall received was 268–619 mm (S1 Fig).

### Plant cultivation

Planting materials used in this study were seeds acquired from the commercial supplier Trade Winds Fruit, Windsor, California. The germinated two weeks old (with 2 true leaves) seedlings were transferred into the polybags (33 cm × 17 cm × 12 cm) filled with mixed topsoil, sand and compost at ratio 2:1:1 v/v. The polybags were kept under partial sunlight shade for three months and thereafter placed under direct sunlight and allowed them to grow before transplanted in the field. The plants were regularly watered. For planting of *P*. *quadrangularis*, a vertical trellis system with twenty rows, each with 25 m was constructed. The trellis system consisted of 2.0 m tall post set at 5 m intervals along the rows. Four months old seedling with similar height (~ 100 cm) and number of leaves (approximately 20–25) were chosen for transplanting with planting distance of 2.0 m between rows and 2.5 m within rows.

### Experimental design and fertilizer management

The experiment was conducted in two cultivation practices: organic cultivation (100% chicken organic fertilizer) and conventional cultivation (100% inorganic fertilizer) both with total of 60 plants. Each treatment was repeated in three blocks and each block composed by 10 plants. Plant management including irrigation, pruning, weeding and pest and disease control were performed accordingly. Fertilizer needs are directly related to the type and nutrient status of the soil. Based on the soil analysis, the soil needs extra nutrients. The application rate for inorganic fertilizer was 250-50-80 kg N P_2_O_5_-K_2_O ha^-1^ and for organic fertilizer was 20 t ha^-1^. Fertilizer application was done each plant every 4 weeks throughout the growing season. The fertilizer applied were within the recommended doses by Olermo et al. [22]. for cultivation of passion fruit.

### Sample collections and preparation

70 kilograms (≃ 50 fruits) vine-ripened *P*. *quadrangularis* fruits (58–60 days after anthesis) were harvested randomly from thirty plants for each treatment at the passion fruit farm, UPMKB, Sarawak during the major harvesting period. The fruits were brought to the laboratory and immediately inspected and cleaned with distilled water. Fruits were dissected into half and the pulps were separated from the mesocarp. The pulps were mechanically separated from the seeds. The pulps and mesocarps were divided into two divisions. Firstly, the fresh extracted pulps and mesocarps were stored at -20°C for determination of physicochemical properties and vitamin content. Secondly, the samples were freeze dried at -47°C with 20 mTorr using ilShin Freeze Dryer System TFD5503 for 3 days. The freeze-dried samples were used for extraction to determination of antioxidant analyses and metabolites profiling. Additionally, the fresh leaves samples collected and brought to the laboratory, immediately inspected and cleaned with distilled water. The leaves were freeze-dried prior to analysis. All the dried parts were ground using a blender unit to a fine powder and stored in airtight containers kept in a desiccator until used for the further analyses on determination of physicochemical and secondary metabolites.

### Physicochemical properties

The physicochemical properties of the organic and conventional fruits were determined using the standard methods of the Association of Official Analytical Chemists [23]. The pH was measured by using a pH meter (method 964.24, [23]) while the total soluble solid was analysed using a handheld pocket refractometer (method 983.17, [23]). Determination of total acidity was done following the titration method (method 942.15, [23]). Ascorbic acid was determined by Indophenol titration method (method 974.29, [23]) and vitamin A, B and E using the HPLC methods (method 974.29, [23]).

### Sample extraction

The sample extraction was performed based on the described method by Ramaiya et al. [24]. Each of the samples was weighed 10 g and extracted with 100 mL of 80% methanol for 3 days. The extraction was carried out with the aid of orbital shaker set at 160 rpm to further facilitate the extraction. The samples were then centrifuged at 500 × *g* for 10 min and the supernatant were filtered through Whatman No. 2 filter paper. Excess solvents were evaporated using rotary evaporator. Crude sample extracts were kept in 4°C prior to the analyses.

### Antioxidant activities

#### 2.7.1 2,2-Diphenyl-1-picrylhydrazyl (DPPH) free radical scavenging activity

Total antioxidant activity was determined using the DPPH method based on quantification of free radical scavenging activity of the extracts described by Brand-Williams et al. [25]. The absorbance of sample was measured at 517 nm using PerkinElmer Lambda 25 UV/VIS Spectrophotometer. The concentration of samples required to scavenge 50% DPPH (EC_50_) was determined by linear regression for the concentration and EC_50_ (%). The lower the EC_50_ value, the higher the antioxidant activity. DPPH radical scavenging activity expressed as mg mL^-1^.

#### Ferric reducing antioxidant power (FRAP) assay

The determination of ferric reducing antioxidant power of the extracts was carried out using FRAP assay followed the modified method of Benzie and Strain [26]. Trolox was used as a standard and TPTZ working reagent used as a blank reference. The absorbance of samples and standards were read at 595 nm using PerkinElmer Lambda 25 UV/VIS Spectrophotometer. Higher optical density indicated higher reducing power. The results were expressed in mg trolox (TE) equivalent 100 g^-1^ dried extract.

#### Total phenolic content (TPC)

The total phenolic content (TPC) in the samples were determined according to Ramaiya et al. [24]. with slight modification by using Folic-Ciocalteu’s reagent spectrophotometrically. The absorbance was measured with an UV-VIS Spectrophotometer at 765 nm. Quantification of TPC was performed using a calibration curve prepared with gallic acid standard. Analysis was performed in triplicate and the results were expressed as mg gallic acid (GAE) equivalent 100 g^−1^ dried extract.

#### Total flavonoid content (TFC)

Total flavonoid content (TFC) was determined by using UV-VIS Spectrophotometer at the wavelength of 510 nm [27]. The absorbance was measured against the blank at 510 nm with an UV-VIS spectrophotometer. A calibration curve was being constructed using standard quercetin and the total flavonoid content was expressed as mg of quercetin (QE) equivalents per 100 g^−1^ dried extract.

### Metabolites profiling

#### Sample extraction

The freeze dried leaves and fruits samples were extracted with 100% methanol in 1:10 (w/v) by sonication water bath. The extracts were then centrifuged and the supernatant were pass through 0.22 um PTFE syringe filter. Further cleanup with Oasis Prime HLB cartridge was applied on the extracts by pass through method (leaves) and wash and elute method (mesocarp and pulp). The extracts were then vacuum dried and reconstitute in 200 uL mobile phase. A pooled sample was prepared by mixing all the samples in a single vial.

#### UPLC-QToF analysis

The prepared extracts (1 uL) were then injected into Waters ACQUITY H-Class UPLC coupled with Xevo G2-XS QToF mass spectrometer for metabolites profiling using MS^e^ technique. Pooled samples was injected after every 10 injections of samples. MS^e^ technique allowed alternating application of low (3 V) and high ramping collision energy (15–45 V) to enable the collection of parents and fragments mass in single injection. Chromatography separation was conducted using Waters ACQUITY HSS T3 (2.1 × 100 mm, 1.8 um) maintained at 40°C at flow rate of 0.3 mL min^-1^. The mobile phase used was A: Water with 0.1% formic acid and B: Acetonitrile with 0.1% formic acid with the gradient profile of 0–5 minutes-10% B; 5–12 minutes-30% B; 12–17 minutes-70% B; 17–20 minutes-90% B; 20–21 minutes-100% B and 21–23 minutes: 10% B. The data were collected in both positive and negative electrospray ionization mode in the range of m/z 50–1000 at scan time of 0.1 s. The mass spectrometer settings applied was capillary voltage at 2.0 kV, source temperature of 120°C, desolvation temperature of 600°C; cone gas of 50 L h^-1^ and desolvation gas of 1000 L h^-1^.

### Statistical analysis

Mean, standard deviation and range were computed for triplicate determination. The data for pH, total soluble solid (TSS) (⁰Brix), total acidity, vitamins, DPPH, FRAP, TPC and TFC contents were statistically analysed using SAS Window Programme 9.4. Independent t-test was used to detect significant differences among the mean comparison between the two treatments.

The raw data files collected from UPLC-QToF instrument using Masslynx Version 4.2 software were then imported into Progenesis QI 2.0 software for peak picking, peak alignment and response normalization. Principal component analysis (PCA) was conducted to evaluate the pooled sample to be tightly clustered and the degree of variation between samples from organic and conventional cultivation. OPLS-DA and binary comparison was later performed between organic treated and control samples using EZInfo 3.0 software between organic treated and control samples. The S-plot with covariance p[1] and correlation p[corr] was generated for visualization of differences and selection of potential markers. The metabolites from S-plot with correlation, p[corr] > 0.9 were then transfer to Progenesis QI 3.0 software for screening of potential discrimination markers.

Progenesis QI 2.0 enables the library matching of the potential markers based on the accurate mass of precursor and also MS/MS fragments obtained from MS^e^ raw data. The mass spectra libraries used in this study were NIST MS/MS library and Waters METLIN MS/MS library, which contain neutral mass, experimental MS/MS and in-silico MS/MS fragments for commonly adducted forms of compounds. The potential marker list was then shortlisted based on the compounds matched with [M+H] or [M-H] adducts from library search against NIST and METLIN MS/MS libraries. Statistical filters of *p* < 0.05, max fold change > 2 and minimum coefficient of variation < 30 were applied. Top candidates from library matches were assigned tentatively to represent the features. The abundance of features elevated in samples from organic cultivation with potential matched identity belong to classes of phenolics, flavonoids, terpenoids and alkaloids were presented by hierarchical clustering technique and visualized in heatmap using Metaboanalyst software (www.metaboanalyst.ca). Correlation analysis (CA) was carried out using XLSTAT software version 2016 (Addinsoft, New York, USA) to obtain the relationship between antioxidant activities and the putatively identified metabolites in samples of organic and conventional cultivations.

## Results and discussion

### Physicochemical properties of organic and conventional fruits of *Passiflora quadrangularis*

Table 1 describes the physicochemical properties of the *P*. *quadrangularis* fruits cultivated under organic and conventional practices. There were no significant difference (*p* > 0.05) between pH and TTA of organic and conventional pulp and mesocarp of *P*. *quadrangularis*. This is contradicting to the findings by Janzantti et al. [14]. where organic cultivation yielded higher TTA (4.32 g citric acid 100 mL^-1^) in *P*. *edulis* Sims. The present finding corroborates previous findings that there is no significant change in total acidity of tomatoes despite different fertilizer applications [28]. The TSS was significantly higher in the pulp and mesocarp of conventional cultivation (14.17 ± 0.03 and 5.83 ± 0.03 ^o^Brix, respectively) compared to organic practice (15.33 ± 0.02 and 6.23 ± 0.02 ^o^Brix, respectively). The conventional passion fruit pulp showed a higher TSS value at 14.71 ^o^Brix [12]. Testing the ^o^Brix level of fruit gives an ideal fruit quality; higher ^o^Brix means better flavour. The ^o^Brix in pulp from *P*. *quadrangularis* is higher (> 14.00 ^o^Brix) compared to the mesocarp (> 5.00 ^o^Brix). The variables examined in this study were within the range of Brazilian Legislation standards e.g. 11 ^o^Brix and pH values between 2.7 and 3.8 to meet the quality of passion fruit pulp.

**Table 1:** Physicochemical properties of organic and conventional fruits of *Passiflora quadrangularis*.

Variables		Organic cultivation	Conventional cultivation
pH	Pulp	3.73 ± 0.03^a^	3.72 ± 0.02^a^
	Mesocarp	5.15 ± 0.02^a^	5.14 ± 0.01^a^
Total titratable acidity (TTA) (%)	Pulp	1.06 ± 0.02^a^	1.04 ± 0.04^a^
	Mesocarp	0.04 ± 0.01^a^	0.04 ± 0.01^a^
Total soluble solid (TSS) (^o^Brix)	Pulp	14.17 ± 0.03^b^	15.33 ± 0.02^a^
	Mesocarp	5.83 ± 0.03^b^	6.23 ± 0.02^a^
^o^Brix/acid	Pulp	13.37	14.74
	Mesocarp	145.75	155.75
Vitamin A (I.U.)	Pulp	871.00 ± 6.43^a^	779.33 ± 8.41^b^
	Mesocarp	244.00 ± 7.21^a^	235.00 ± 5.97^a^
Vitamin C (mg 100 g^-1^ FW)	Pulp	22.04 ± 0.37^a^	19.44 ± 0.81^b^
	Mesocarp	12.13 ± 0.15^a^	10.17 ± 0.10^b^
Vitamin B2 (mg 100 g^-1^ FW)	Pulp	0.11 ± 0.02^a^	0.13 ± 0.01^a^
	Mesocarp	0.32 ± 0.01^a^	0.31 ± 0.01^a^
Vitamin B3 (mg 100 g^-1^ FW)	Pulp	0.97 ± 0.05^a^	0.82 ± 0.02^b^
	Mesocarp	1.45 ± 0.02^a^	1.23 ± 0.02^b^

Mean values in the same row (organic versus conventional) are significantly different at *p* < 0.05 (Independent t-test). Values are given in means ± standard error.

Vitamins are organic compounds that required in the diet in small amount to maintain normal health and metabolic integrity [29]. Four vitamin groups were identified and quantified in *P*. *quadrangularis* fruits; i.e, vitamin A, B2, B3 and C. Vitamin A and C were higher in the pulp, whereas the mesocarp showed higher vitamin B2 and B3 contents (Table 1). There was a significant difference between the vitamin contents of A, B3 and C in organically grown fruit suggests that the cultivation system influences vitamin content production. Similar findings on vitamin C content were recorded in organically treated *P*. *edulis* [14, 30] and also *Capsicum chinenses* compared to the synthetic fertilizer [31]. This may be due to the differences in composition between organic and inorganic fertilizers and their effects on soil ecology and plant metabolism [32]. The major vitamin in the pulp of *P*. *quadrangularis* was vitamin A ranged 871.00 ± 6.43 I.U. (organic) and 779.33 ± 8.41 I.U. (conventional). One distinctive quality of the passion fruit is the high vitamin C content. The vitamin C content was significantly higher in pulp of organic fruits (22.04 ± 0.37 mg 100 g^-1^ FW) and the mesocarp (12.13 ± 0.15 mg 100 g^-1^ FW). According to the Natural Food Hub, any food with 15–30 mg ascorbic acid can be considered a very good source of ascorbic acid. It is generally accepted that vitamin C levels in fruits are subject to a wide range of environmental factors, i.e., light, temperature, salts, atmospheric contaminants, metals, and herbicides [33–35]. This variable nature could explain the differences between the fruit produced via the two evaluated cultivation systems assessed in this work. This may be due to differences in composition between organic and inorganic fertilizers and their effects on soil ecology and plant metabolism [32].

### Antioxidant activities of *Passiflora quadrangularis*

The results for total antioxidant activities; DPPH, FRAP, TPC and TFC of the leaves, pulp and mesocarp of organically and conventionally grown *P*. *quadrangularis* are shown in Table 2. The antioxidant activities of *P*. *quadrangularis* plant parts varied with cultivation system. Among the plant parts of *P*. *quadrangularis* the leaves possessed higher antioxidant activities, followed by mesocarp and pulp. The TPC value was higher in the leaves and mesocarp under organic fertilization that recorded 174.76 ± 1.13 mg GAE 100 g^-1^ and 64.48 ± 1.51 mg GAE 100 g^-1^, respectively. Contrarily, no significant differences obtained in organic and conventional pulp which was 27.60 ± 0.75 mg GAE 100 g^-1^ and 23.84 ± 1.42 mg GAE 100 g^-1^, respectively. The leaves possessed higher TFC content than the TPC. Leaves of different amaranth species also had higher TPC, TFC and DPPH antioxidant capacity [36, 37].

**Table 2:** Antioxidant activities of *Passiflora quadrangularis* plant parts based on the organic and conventional cultivation practices.

Variables	Treatment	Leaves	Mesocarp	Pulp
TPC (mg GAE 100 g^-1^)	Organic	174.76 ± 1.13^a^	64.48 ± 1.51^a^	27.60 ± 0.75^a^
	Conventional	150.30 ± 0.45^b^	54.47 ± 0.74^b^	23.84 ± 1.42^a^
TFC (mg QE 100 g^-1^)	Organic	3554.39 ± 1.74^a^	39.14 ± 2.62^a^	29.14 ± 0.50^a^
	Conventional	2795.31 ± 2.84^b^	34.64 ± 1.30^a^	25.89 ± 0.13^b^
TAA (DPPH) (mg mL^-1^)	Organic	2.31 ± 0.02^b^	4.88 ± 0.13^b^	13.61 ± 0.34^b^
	Conventional	4.69 ± 0.17^a^	6.87 ± 0.32^a^	18.41 ± 0.58^a^
TAA (FRAP) (mg TE 100 g^-1^)	Organic	165.19 ± 1.22^a^	159.93 ± 0.62^a^	113.24 ±1.97^a^
	Conventional	140.22 ± 1.21^b^	151.02 ± 2.98^a^	94.04 ± 2.26^b^

Mean values in the same column (organic versus conventional) are significantly different at *p* < 0.05 (Independent t-test). Values are given in means ± standard error.

Organic leaves of *P*. *quadrangularis* showed higher TFC value ranged 3554.39 ± 1.74 mg QE 100 g^-1^. Significantly higher TFC value was also recorded in pulp from organic cultivation (29.14 ± 0.50 mg QE 100 g^-1^). It is therefore conceivable that the result of this study showed that the *P*. *quadrangularis* cultivated in organic system, producing higher phenolic and flavonoid contents in comparison to conventional cultivation. The same trend was observed in organically produced fruits, e.g., passion fruit [14], raspberries [15] and strawberry [38]. Several findings have linked the accumulation of phenolics and flavonoids to the level of available N in the soil [39]. The increases in secondary metabolites and antioxidants in organic fertilizer may have been caused by the availability of various other major and minor elements, whereas the chemical fertilizer supplied only the three principal elements, i.e., N, P, and K. This could improve the nutrient accessibility and physiological functions that may enhance the metabolic pathways, including ones which synthesise secondary metabolites compounds, which closely linked with photosynthetic cycle [9, 40].

Additionally, the stronger antioxidant activity was observed in leaves of organic cultivation (2.31 ± 0.02 mg mL^-1^) compared to conventional practice (4.69 ± 0.17 mg mL^-1^). Similarly, the organic mesocarp and pulp possessed stronger DPPH values with 4.88 ± 0.13 mg mL^-1^ and 13.61 ± 0.34 mg mL^-1^, respectively. Higher FRAP value was recorded in organic cultivation for leaves (165.19 ± 1.22 mg TE 100 g^-1^) and pulp (113.24 ± 1.97 mg TE 100 g^-1^) but, mesocarp does not show significant differences compared to the conventional practice. The higher level of TPC, TFC, DPPH and FRAP in organic indicated that cultivation practices influenced the antioxidant bioactive compounds in *P*. *quadrangularis*. Among the plant parts, the leaves contained ample of bioactive compounds that enhanced by the organic cultivation contributed to the high antioxidant activities. Numerous studies have explained that plants tend to produce more or higher level of bioactive compounds and antioxidant as a preventive or protective measure against oxidative and abiotic stress which may result from low level or slow release of macronutrient like N [41–43]. Application of organic fertilizer may create oxidative stress from superoxide dismutase which is a key enzyme in plant defense and development, and abiotic stress which causes accumulation of reactive oxygen species (ROS) that inhibits enzymatic activity, disturbs cellular homeostasis and ruptures membrane with deleterious effects on plant growth [44, 45]. Consequently, organically grown plants react to this conditions by activating their own defense mechanisms which leads to the synthesizing of more bioactive and antioxidant compounds [41]. They activate the signalling pathway responsible for the detoxification of ROS by synthesizing antioxidants that scavenges ROS [46–49].

### Comparison of metabolites profiles between organic and conventional cultivation

**Fig 1:**
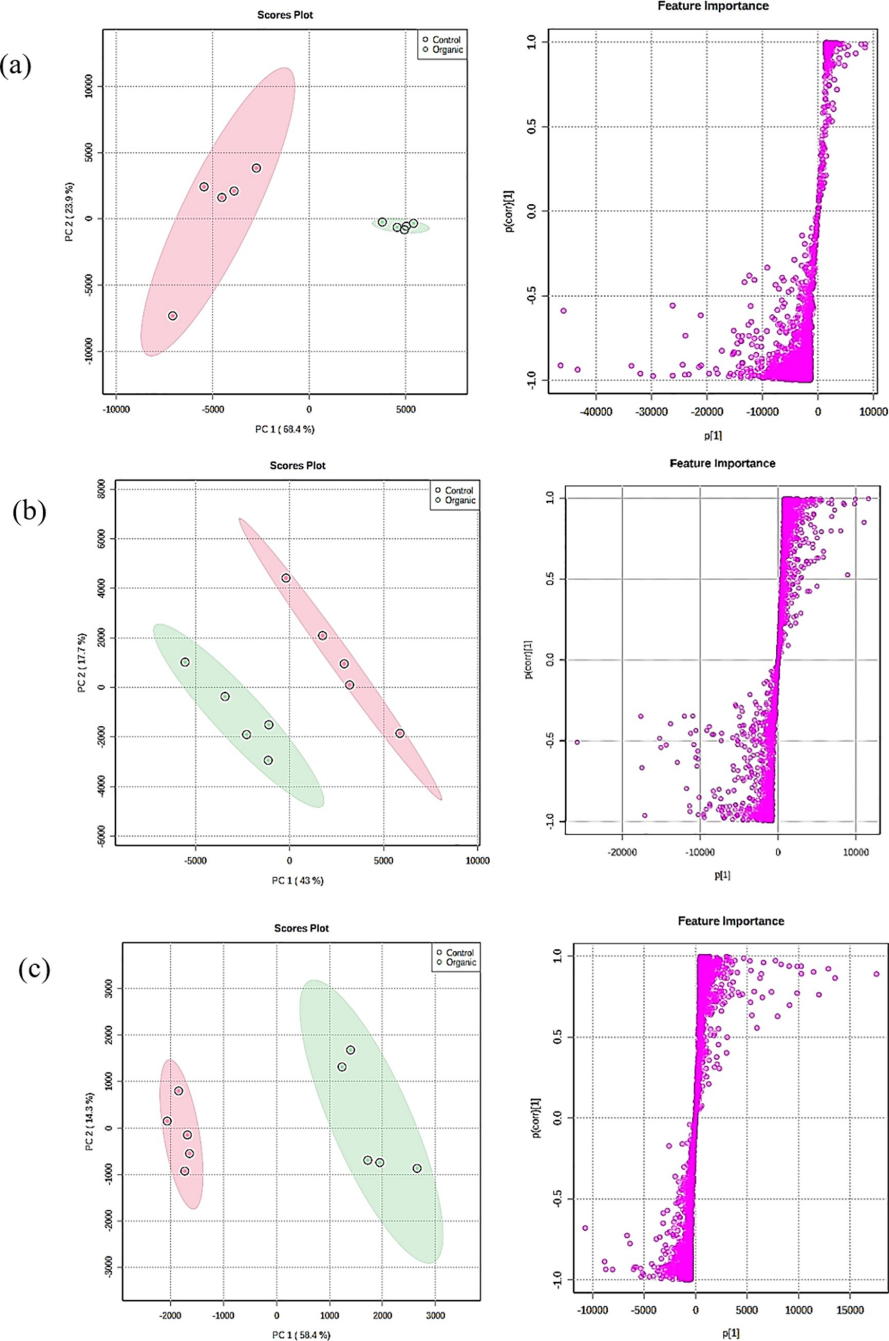
PCA score plots and OPLS-DA S-plots of metabolites analyzed by UPLC-QToF in (a) leaves, (b) mesocarp and (c) pulp of *Passiflora quadrangularis*.

Metabolomics based on mass spectrometer is a powerful technique which combined metabolites profiling and chemometrics to identify metabolites responsible for biological responses [50, 51]. Increasing application in plant science to understand the phytochemical composition and responses to abiotic stresses were reported [44, 52, 53]. In this study, principal component analysis of metabolites profiles revealed that *P*. *quadrangularis* responded to organic and conventional practices with distinct chemical profiles in various plant parts. Based on two vectors in 2D space of principal component analysis (Fig 1), variance of 92.3%, 60.7% and 72.7% could be explained in leaves, mesocarp and pulp respectively in positive ionization mode. Different ionization modes are known to ionize different chemical compounds, the metabolites profiles based on negative ionization on the samples showed variance of 86.7%, 67.8% and 76.2% for leaves mesocarp and pulp samples. These showed that the effect of organic practices on metabolites profile was largest in leaves, followed by pulp and mesocarp. The distinct clustering of the samples between organic and conventional practices prompted for further analysis into the differential metabolites in leaves, mesocarp and pulp. OPLS-DA models (Fig 1) were built for mesocarp, pulp and leaves between organic and chemical treatments and showed good R2 (> 98%) and prediction of goodness, Q2 (> 95%). The metabolites with correlation p(corr)[1] > 0.9 for samples treated with organic fertilizer were selected from S-plots (Fig 1) for markers elucidation.

### Secondary metabolites elevated in *Passiflora quadrangularis* under organic cultivation

**Fig 2:**
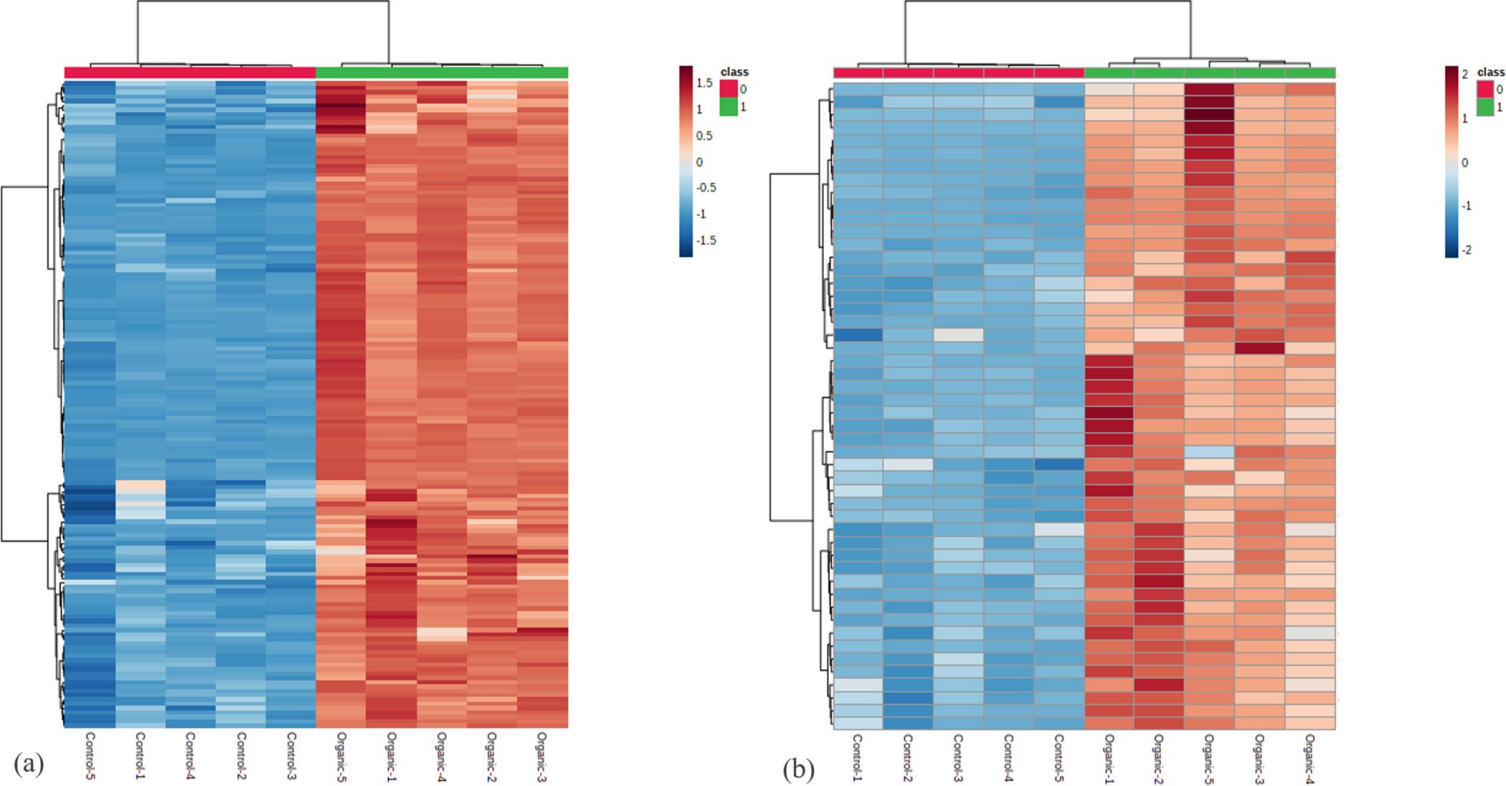
Comparison of the abundance of secondary metabolite between organic (1) and control (0) in *Passiflora quadrangularis* (a) leaves (b) mesocarp and pulp. The samples represented by each box at x-axis and the metabolites were represented by each box at y-axis. The analysis is based on normalized abundance and scaled by Pareto scaling using Metaboanalyst software.

The MS^e^ data acquisition supplied a structural information that can be used for compound identification. The shortlisted metabolites list from S-plots were statistically filtered based on ANOVA (*p* < 0.05), max fold change (> 2) and minimum coefficient of variation (< 30%) and assigned with tentative identification based on primary adducts of [M+H] and [M-H] from MS/MS library matching. A total of 197 putatively identified secondary metabolites (S1 and S2 Tables) were detected and showed higher abundance in leaves (Fig 2A) and edible parts (Fig 2B) of *P*. *quadrangularis* in organic cultivation. This finding is supported by previous studies which have shown organic plant produced higher phenolics and flavonoids content [9, 39, 40, 54, 55]. Janzantti et al. [14]. also had reported organic cultivation influence the aroma and antioxidant activity of passion fruits which related to secondary metabolism. *Passiflora* family has been reported to be rich sources of secondary metabolites. Flavonoids glycosides, cyanogenic glycosides and harmine alkaloids were identified to be the major secondary metabolites in *Passiflora* leaves and possess bioactive properties [21, 52, 56]. The metabolites profiles have been reported varied with species, variants and cultivation system [38, 57]. Further elucidation on the secondary metabolites in the extracts of *P*. *quadrangularis* leaves and edible parts (mesocarp and pulp) from organic cultivation were conducted.

In this study, 149 putatively identified secondary metabolites were detected in leaves extract (S2 Table) had showed elevated abundance level from organic cultivation. The secondary metabolites in leaves are mainly flavonoids derivatives, phenolics, terpenoids. Among these secondary metabolites, 23 metabolites showed more than 5-fold abundance increment in organic compared to conventional practices. Four putatively identified flavonoids and its derivatives which had similarly reported in *Passiflora* sp. namely isoquercitin, myricetin, isovitexin 2’’-O-(6‴-(E)-P-Coumaroyl), Glucoside 4’-O-Glucoside, 3,6,3’,4’-tetrahydroxyflavone are among the prominent metabolite markers [56, 58]. The chemical marker of *P*. *quadrangularis* (vitexin-2”-O-xyloside) reported by Costa et al. [59]. has shown 3-fold abundance increment from organic cultivation in this study. The cyclopassifloside saponins reported by Sakalem et al. [60]. in the leaves of *P*. *quadrangularis* were not elevated from organic cultivation in leaves sample of this study.

To our knowledge, the metabolites profile of mesocarp and pulp of *P*. *quadrangularis* has not been reported. Flavonoid derivatives, phenolics and terpenoids were the major classes of the secondary metabolites in edible parts (S1 Table) similar to leaves (S2 Table). Nevertheless, the chemical composition of mesocarp is different from pulp and leaves whereby phenolics appeared to be the dominant chemical species with coumarins and cinnamic acids as the major phenolic compounds. Cyclopassifloside saponins reported in leaves of *Passiflora* sp. by Sakalem et al. [60]. was detected and elevated at about 4-fold in the mesocarp from organic cultivation. Norharman alkaloids, the common alkaloids present in *Passiflora* species [21] was detected in the mesocarp and showed 2.5-fold increment of abundance in organic cultivation.

A phenolic compound putatively identified as 2-isopropyl-3-methoxycinnamic acid are shown to be common markers for pulp and mesocarp with 6-fold and 10-fold enhancement respectively in samples from organic cultivation. The pulp has the dominant chemical species from the class of flavonoids derivatives. Myricetin isom and malvidin-3-O-arabinoside isomers are the common chemical markers that elevated in organic cultivation in pulp and leaves. This study had reported for the first time the difference in secondary metabolites between mesocarp and pulp of *P*. *quadrangularis* and provide insights at compound level on its potential consumption benefits.

### Correlation between prominent secondary metabolites markers and antioxidant activities

The contribution of secondary metabolites to antioxidant or bioactivity has been reported [21, 56, 61]. In this study, the prominent secondary metabolites identified with maximum fold change more than 5 were then correlate to the antioxidant activities of respective samples to understand the contribution of these metabolites to different antioxidant activities. Good correlation between abundance of prominent markers to various antioxidant activities were obtained. Three prominent markers in pulp samples (Table 3), putatively identified as eriocitrin, terminaline and isopropyl methoxycinnamic acid were well correlated (r > 0.85) with total flavonoids content, DPPH scavenging activity and FRAP. Meanwhile, the prominent markers in mesocarp samples (Table 3), putatively identified as dihydroxymethylcoumarin, mescaline, theasapogenol, hydroxycadalene and isopropyl cinnamic acid showed good correlation (r > 0.95) with total phenolics content and DPPH scavenging activity. The 23 shortlisted prominent markers in leaves samples were correlated with all antioxidant activities evaluated (Table 4). These results suggested that mesocarp of *P*. *quadrangularis* are phenolics rich and possess good antioxidant properties by the mechanism of radical scavenging activity. The pulp of *P*. *quadrangularis* are flavonoids rich and contributed to radical scavenging and ferric reducing power. The leaves of *P*. *quadrangularis* showed good composition of phenolics, flavonoids and terpenoids with outstanding antioxidant activities among different plant parts. The correlation of leaf phenolics and flavonoids with DPPH antioxidant capacity were corroborative to different species of *Amaranthus* leafy vegetables [10, 62, 63].

**Table 3:** Pearson correlation coefficients of antioxidant activities and prominent markers in fruit parts of *Passiflora quadrangularis*.

RT (min)	m/z	Adducts	MS/MS fragments	Putatively Identified Markers	Total Phenolics Content	Total Flavonoids Content	DPPH Scavenging Activity	Ferric Reducing Antioxidant Power
**Pulp**								
3.77	595.1679	M-H	151.0040, 287.0566	Eriocitrin	0.44	**0.96**	**-0.96**	**0.87**
11.08	364.3221	M+H	-	Terminaline	0.71	**0.91**	**-0.87**	**0.97**
13.54	221.1181	M+H	131.0860, 143.0860, 105.0700	Isopropyl methoxycinnamic acid	0.63	**0.91**	**-0.97**	**0.94**
**Mesocarp**								
1.58	215.0316	M+Na	175.0366, 151.0390, 147.0435	Dihydroxy methylcoumarin	**0.96**	0.65	**-0.95**	0.66
4.29	212.1283	M+H	131.0497, 115.0544, 91.0543	Mescaline	**0.95**	0.62	**-0.94**	0.66
8.26	507.3681	M+H	-	Theasapogenol	**0.95**	0.61	**-0.94**	0.66
8.33	215.1436	M+H	158.0733, 128.0616, 115.0538	Hydroxycadalene	**0.96**	0.67	**-0.94**	0.66
13.54	221.1181	M+H	131.0860, 143.0860, 105.0700	Isopropyl methoxycinnamic acid	**0.94**	0.67	**-0.90**	0.66

All values in bold are significantly different at *p* < 0.05. Markers were putatively identified on the basis of accurate mass, MS/MS fragmentation and isotope similarity using MS^e^ and isotope distribution data which matched with NIST and METLIN MS/MS libraries using Progenesis QI 2.0.

**Table 4:** Pearson correlation coefficients of antioxidant activities and prominent markers in leaves of *Passiflora quadrangularis*.

RT (min)	m/z	Adducts	MS/MS fragments	Putatively Identified Markers	Total Phenolics Content	Total Flavonoids Content	DPPH Scavenging Activity	Ferric Reducing Antioxidant Power
1.86	300.1807	M+H	138.0910, 98.0600, 70.0651	Isolycopsamine	**1.00**	**1.00**	**-0.99**	**0.99**
1.93	138.0913	M+H	138.0910, 108.0810, 79.0542	Methyridine	**0.97**	**0.96**	**-0.95**	**0.97**
11.15	397.3106	M+H	81.0699, 93.0699, 107.0857	Delta tocotrienol	**0.99**	**1.00**	**-0.99**	**0.99**
13.47	392.1329	M+H	-	Glycoperine	**0.97**	**0.98**	**-0.96**	**0.97**
14.93	443.2057	M+H	-	Exiguaflavanone	**0.98**	**0.99**	**-0.97**	**0.98**
19.21	205.1236	M+H	97.1012	Butylcinnamate	**0.95**	**0.96**	**-0.94**	**0.96**
2.13	393.2102	M+H	-	Dexamethasone	**0.96**	**0.93**	**-0.91**	**0.95**
2.34	303.0505	M+H	-	Pentahydroxyflavone	**1.00**	**1.00**	**-0.99**	**1.00**
2.34	465.1039	M+H	353.0520	Isoquercetin	**1.00**	**1.00**	**-0.99**	**1.00**
2.36	237.1602	M+H	159.0925, 132.0817	Dropropizine	**1.00**	**0.98**	**-0.97**	**0.99**
2.59	903.2548	M+H	427.1020, 283.0600	Isovitexin glucoside	**0.96**	**0.98**	**-0.98**	**0.97**
2.73	175.0765	M+H	128.0620, 115.0543, 91.0541	Dimethylchromone	**0.99**	**1.00**	**-0.99**	**0.99**
3.53	319.0450	M+H	273.0400	Myricetin	**0.99**	**0.99**	**-0.98**	**0.99**
3.82	186.0553	M+H	89.0388, 77.0389	Cyanomethylchromone	**0.96**	**0.95**	**-0.94**	**0.95**
4.04	186.0550	M+H	158.0598, 115.0542	Cyanomethylchromone	**0.99**	**0.99**	**-0.98**	**0.98**
4.04	195.0653	M+H	177.0550, 149.0600	Isoferulic acid	**0.90**	**0.92**	**-0.91**	**0.89**
4.35	630.3331	M+H	-	Falaconitine	**0.96**	**0.97**	**-0.95**	**0.95**
6.29	291.1048	M+H	247.0798	Furaneol glucoside	**0.99**	**1.00**	**-0.99**	**0.99**
7.00	545.3843	M+H	439.3570, 95.0855	Ganoderic acid	**0.99**	**0.99**	**-0.98**	**0.99**
7.70	287.0559	M+H	213.0550, 123.0443	Tetrahydroxyflavone	**0.98**	**0.99**	**-0.98**	**0.97**
9.59	487.3420	M+H	-	Quillaic acid	**1.00**	**1.00**	**-0.99**	**0.99**
9.68	487.3418	M+H	-	Quallaic acid	**0.99**	**1.00**	**-0.99**	**0.99**
9.90	927.4819	M+H	-	Tragopogonsaponin	**0.99**	**0.99**	**-0.98**	**1.00**

All values in bold are significantly different at *p* < 0.05. Markers were putatively identified on the basis of accurate mass, MS/MS fragmentation and isotope similarity using MS^e^ and isotope distribution data which matched with NIST and METLIN MS/MS libraries using Progenesis QI 2.0.

## Conclusion

In summary, the present work confirms that agronomic factors may have a significant impact on the antioxidant activities and secondary metabolites in *P*. *quadrangularis*. Organic cultivation enhanced the antioxidant activities and accumulation of secondary metabolites particularly flavonoids in the leaves and pulp, while phenolics in mesocarp. There are potential health benefits by direct consumption of mesocarp and pulp of *P*. *quadrangularis* from organic cultivation. The leaves as by-product possessed strongest antioxidant activities with the high abundance and diversity of secondary metabolites suggested its wide potential for application in pharmaceutical or nutraceutical industries.

## Supporting information

S1 Fig Meteorological data for monthly rainfall and temperature at Bintulu from January 2019 to March 2020. (DOCX)

S1 Table Secondary metabolites that elevated in edible parts of *Passiflora quadrangularis* treated with organic cultivation. (DOCX)

S2 Table Secondary metabolites that elevated in young leaves of *Passiflora quadrangularis* treated with organic cultivation. (DOCX)
