# Supplementary material for: Organic cultivation practices enhanced antioxidant activities and secondary metabolites in giant granadilla (Passiflora quadrangularis L.)
Source: PLoS One. 2021 Jul 26;16(7):e0255059. doi: 10.1371/journal.pone.0255059 (PMC8312946; doi:10.1371/journal.pone.0255059)
Supplement: S1 Fig — (DOCX) [file pone.0255059.s001.docx]

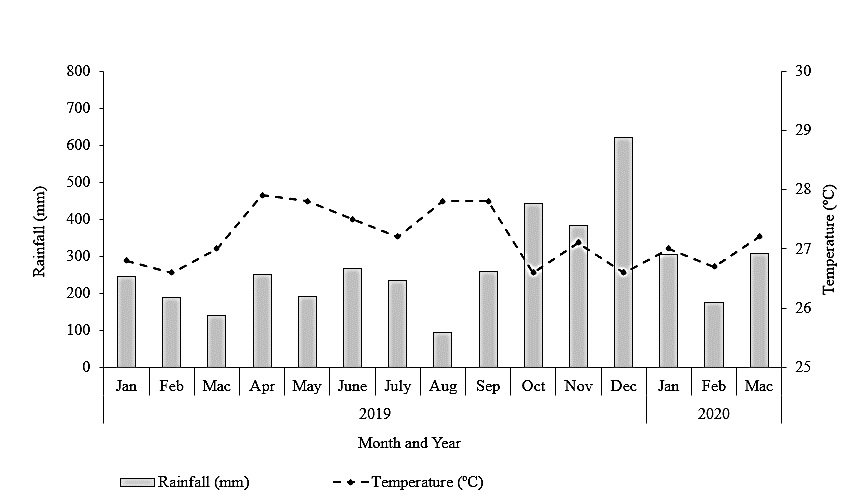


**S1 Fig. Meteorological data for monthly rainfall and temperature at Bintulu from January 2019 to March 2020.**
