# Supplementary material for: Organic cultivation practices enhanced antioxidant activities and secondary metabolites in giant granadilla (Passiflora quadrangularis L.)
Source: PLoS One. 2021 Jul 26;16(7):e0255059. doi: 10.1371/journal.pone.0255059 (PMC8312946; doi:10.1371/journal.pone.0255059)
Supplement: S1 Table — (DOCX) [file pone.0255059.s002.docx]

**S1 Table. Secondary metabolites that elevated in edible parts of *Passiflora quadrangularis* treated with organic cultivation.**

| **RT (min)** | **m/z** | **Adducts** | **Formula** | **Max fold change** | **Mass Error (ppm)** | **Isotope Similarity** | **Tentative Assignment** | **Parts** |
| --- | --- | --- | --- | --- | --- | --- | --- | --- |
| 1.18 | 395.0948 | M+H | C_18_H_18_O_10_ | 4.82 | -6.21 | 95.60 | 9-Hydroxy-4-Methoxypsoralen 9-Glucoside | Mesocarp |
| 1.47 | 457.1886 | M+H | C_25_H_28_O_8_ | 1.98 | 6.29 | 77.89 | Lupinisoflavone | Pulp |
| 1.58 | 149.0598 | M+K | C_9_H_8_O_2_ | 2.99 | 0.42 | 98.25 | Dihydrocoumarin | Mesocarp |
| 1.58 | 215.0316 | M+Na | C_10_H_8_O_4_ | 5.01 | 0.19 | 98.95 | 7,8-Dihydroxy-4-Methylcoumarin | Mesocarp |
| 1.86 | 357.2042 | M+H | C_22_H_28_O_4_ | 1.98 | -5.17 | 70.68 | Nitenin | Pulp |
| 2.10 | 357.2032 | M+H | C_22_H_28_O_4_ | 2.02 | -8.07 | 88.48 | Gamma-Crocetin | Pulp |
| 2.22 | 163.0764 | M+H | C_10_H_10_O_2_ | 7.79 | 6.24 | 93.34 | Alpha-Methyl Cinnamic Acid | Pulp |
| 2.52 | 391.1881 | M+H | C_25_H_26_O_4_ | 2.27 | -5.79 | 92.82 | Shinflavanone | Pulp |
| 3.00 | 169.0754 | M+H | C_11_H_8_N_2_ | 2.57 | -3.55 | 88.13 | Norharman | Mesocarp |
| 3.05 | 621.1807 | M+H | C_29_H_32_O_15_ | 2.85 | -1.09 | 86.18 | Cratenacin | Pulp |
| 3.24 | 179.0339 | M+H | C_9_H_6_O_4_ | 2.20 | 0.05 | 97.53 | Esculetin | Mesocarp |
| 3.27 | 423.2174 | M+H | C_26_H_30_O_5_ | 1.94 | 1.86 | 87.46 | Nitidulin | Pulp |
| 3.30 | 163.0392 | M+H | C_9_H_6_O_3_ | 2.38 | 1.69 | 97.60 | 7-Hydroxycoumarin | Pulp |
| 3.72 | 441.2228 | M+H | C_26_H_32_O_6_ | 2.41 | -9.94 | 95.43 | 5,7-Dihydroxy-4'-Methoxy-8-C-Prenyl-3'-(3-Hydroxy-3-Methylbutyl)Flavanone | Pulp |
| 3.77 | 595.1679 | M-H | C_27_H_32_O_15_ | 9.1 | 1.85 | 89.10 | Eriocitrin | Pulp |
| 3.83 | 342.2416 | M-H | C_22_H_33_NO_2_ | 4.55 | -6.57 | 85.40 | Atisine | Pulp |
| 3.84 | 319.0460 | M+H | C_15_H_10_O_8_ | 2.18 | 3.71 | 88.73 | Myricetin | Pulp |
| 3.87 | 479.0843 | M-H | C_21_H_20_O_13_ | 3.1 | 2.51 | 88.70 | Myricetin 3-Glucoside | Pulp |
| 3.90 | 405.2032 | M+H | C_26_H_28_O_4_ | 2.26 | -6.96 | 91.84 | Boesenbergin | Pulp |
| 3.98 | 345.1443 | M-H | C_18_H_22_N_2_O_5_ | 2.58 | -3.72 | 86.86 | Coumarin-Suberoylanilide Hydroxamic Acid | Pulp |
| 4.23 | 328.0808 | M+FA-H | C_17_H_15_NO_6_ | 2.77 | -5.54 | 91.49 | 4',6'-Dimethoxy-2'-Hydroxy-3-Nitrochalcone | Pulp |
| 4.29 | 212.1283 | M+H | C_11_H_17_NO_3_ | 9.05 | 0.62 | 98.41 | Mescaline | Mesocarp |
| 4.69 | 163.0402 | M-H | C_9_H_8_O_3_ | 2.03 | 0.58 | 90.09 | Trans-2-Hydroxycinnamic Acid | Pulp |
| 4.94 | 177.0194 | M-H | C_9_H_6_O_4_ | 4.22 | 0.10 | 89.91 | 7,8-Dihydroxycoumarin | Pulp |
| 5.17 | 303.0502 | M+H | C_15_H_10_O_7_ | 2.14 | 1.05 | 97.79 | Delphinidin | Mesocarp |
| Continued | | | | | | | | |
| **RT (min)** | **m/z** | **Adducts** | **Formula** | **Max fold change** | **Mass Error (ppm)** | **Isotope Similarity** | **Tentative Assignment** | **Parts** |
| 5.68 | 257.0830 | M-H | C_15_H_14_O_4_ | 4.24 | 4.24 | 88.68 | Rhapontigenin | Pulp |
| 5.82 | 187.0395 | M+H | C_11_H_6_O_3_ | 2.35 | 2.86 | 93.77 | Angelicin | Pulp |
| 5.85 | 177.0193 | M-H | C_9_H_6_O_4_ | 3.4 | -0.25 | 98.54 | Esculetin | Pulp |
| 5.85 | 343.0833 | M-H | C_18_H_16_O_7_ | 2.78 | 2.96 | 89.98 | 5,3'-Dihydroxy-6,7,4'-Trimethoxyflavone | Pulp |
| 6.01 | 461.1110 | M-H | C_22_H_22_O_11_ | 3.09 | 4.41 | 83.06 | Malvidin-3-O-Arabinoside | Pulp |
| 7.36 | 271.0986 | M-H | C_16_H_16_O_4_ | 3.18 | 3.62 | 87.74 | Angolensin | Pulp |
| 8.25 | 699.4367 | M+H | C_37_H_62_O_12_ | 4.23 | 7.53 | 95.62 | Cyclopassifloside | Mesocarp |
| 8.26 | 151.1118 | M+H | C_10_H_14_O | 3.02 | 0.41 | 98.09 | Carvacrol | Mesocarp |
| 8.26 | 507.3681 | M+H | C_30_H_50_O_6_ | 8.31 | 0.23 | 92.86 | Theasapogenol A | Mesocarp |
| 8.33 | 215.1436 | M+H | C_15_H_18_O | 5.14 | 2.76 | 92.93 | 7-Hydroxycadalene | Mesocarp |
| 8.33 | 251.1636 | M+H | C_15_H_22_O_3_ | 3.32 | -2.16 | 92.81 | Ketosantalic Acid | Mesocarp |
| 8.51 | 251.1619 | M+H | C_15_H_22_O_3_ | 3.76 | -8.99 | 97.63 | Ketosantalic Acid | Mesocarp |
| 9.78 | 441.3726 | M+H | C_30_H_48_O_2_ | 2.73 | -0.23 | 82.89 | Soyasapogenol | Mesocarp |
| 11.08 | 364.3221 | M+H | C_23_H_41_NO_2_ | 6.73 | 3.09 | 89.52 | Terminaline | Pulp |
| 11.37 | 413.2095 | M-H | C_23_H_30_N_2_O_5_ | 3.25 | 3.13 | 85.57 | Desacetylvindoline | Pulp |
| 12.37 | 317.0625 | M+H | C_16_H_12_O_7_ | 2.33 | -9.63 | 82.68 | Quercetin 3'-Methyl Ether | Pulp |
| 12.40 | 315.0503 | M-H | C_16_H_12_O_7_ | 3.17 | -2.39 | 82.68 | 7,8,4"-Trihydroxyisoflavone | Pulp |
| 12.46 | 251.1272 | M+H | C_14_H_18_O_4_ | 2.65 | -2.21 | 96.70 | Ubiquinone | Mesocarp |
| 13.10 | 237.1860 | M+H | C_15_H_24_O_2_ | 2.22 | 4.54 | 95.12 | Curcumol | Mesocarp |
| 13.53 | 471.3486 | M-H | C_30_H_48_O_4_ | 3.01 | 1.29 | 80.61 | Maslinic Acid | Pulp |
| 13.54 | 221.1181 | M+H | C_13_H_16_O_3_ | 10.1 (Mesocarp)  6.66  (Pulp) | 3.98 | 94.28 | 2-Isopropyl-3-Methoxycinnamic Acid | Pulp, Mesocarp |
| 13.98 | 449.1576 | 2M+H | C_15_H_12_O_2_ | 2.44 | -1.33 | 92.24 | 4-Hydroxychalcone | Mesocarp |
| 16.93 | 575.5043 | M+H | C_37_H_66_O_4_ | 2.87 | 1.52 | 97.45 | Montecristin | Pulp |

Peaks were putatively identified on the basis of accurate mass, MS/MS fragmentation and isotope similarity using MS^e^ and isotope distribution data which matched with NIST and METLIN metabolite databases using Progenesis QI 2.0.
