# Supplementary material for: Organic cultivation practices enhanced antioxidant activities and secondary metabolites in giant granadilla (Passiflora quadrangularis L.)
Source: PLoS One. 2021 Jul 26;16(7):e0255059. doi: 10.1371/journal.pone.0255059 (PMC8312946; doi:10.1371/journal.pone.0255059)
Supplement: S2 Table — (DOCX) [file pone.0255059.s003.docx]

**S2 Table. Secondary metabolites that elevated in young leaves of *Passiflora quadrangularis* treated with organic cultivation.**

| **RT (min)** | **m/z** | **Adducts** | **Formula** | **Max fold change** | **Mass Error (ppm)** | **Isotope Similarity** | **Tentative Assignment** |
| --- | --- | --- | --- | --- | --- | --- | --- |
| 1.03 | 241.1545 | M+H | C_12_H_20_N_2_O_3_ | 2.93 | -0.56 | 95.45 | Pirbuterol |
| 1.12 | 501.3035 | M+H | C_26_H_44_O_9_ | 2.46 | -4.67 | 59.29 | Mupirocin |
| 1.20 | 285.0997 | M-H | C_13_H_18_O_7_ | 2.22 | 6.03 | 89.26 | Salicin |
| 1.45 | 189.0405 | M-H | C_7_H_10_O_6_ | 3.85 | -0.03 | 92.80 | 3-Dehydroquinic Acid |
| 1.45 | 297.1334 | M-H | C_16_H_18_N_4_O_2_ | 4.44 | -7.64 | 87.99 | Nialamide |
| 1.63 | 297.1343 | M-H | C_16_H_18_N_4_O_2_ | 3.39 | -4.61 | 89.19 | Nialamide |
| 1.71 | 243.1359 | M+NH4 | C_16_H_18_O_2_ | 2.47 | -8.56 | 94.77 | 14-Methoxy-4,4-Bisnor-4,8,11,13-Podocarpatetraen-3-One |
| 1.72 | 373.1139 | M-H | C_16_H_22_O_10_ | 2.17 | -0.41 | 95.12 | Geniposidic Acid |
| 1.77 | 215.1393 | M+H | C_10_H_18_N_2_O_3_ | 2.92 | 1.34 | 96.73 | D-Dethiobiotin |
| 1.78 | 247.1046 | M+Na | C_11_H_16_N_2_O_3_ | 2.75 | 0.75 | 96.98 | Butalbital |
| 1.79 | 467.1584 | M-H | C_22_H_28_O_11_ | 2.06 | 5.30 | 70.17 | Prim-O-Glucosylcimifugin |
| 1.82 | 275.1047 | M+H | C_14_H_14_N_2_O_4_ | 2.41 | 7.64 | 76.75 | (1xi,3s)-1,2,3,4-Tetrahydro-1-Methyl-Beta-Carboline-1,3-Dicarboxylic Acid |
| 1.84 | 407.1887 | M+H | C_25_H_26_O_5_ | 2.58 | 8.34 | 96.06 | Flemingin D |
| 1.86 | 300.1807 | M+H | C_15_H_25_NO_5_ | 17.40 | 0.61 | 88.52 | Isolycopsamine |
| 1.88 | 243.1341 | M+H | C_11_H_18_N_2_O_4_ | 1.97 | 0.78 | 93.57 | N-Hydroxypentobarbital |
| 1.93 | 138.0913 | M+H | C_8_H_11_NO | 750.00 | -0.31 | 99.14 | Methyridine |
| 1.93 | 755.2356 | M+H | C_34_H_42_O_19_ | 11.80 | -4.88 | 79.50 | Rhamnocitrin 3-Rhamninoside |
| 2.00 | 197.0845 | M+H | C_10_H_13_C_l_N_2_ | 2.48 | 2.66 | 54.74 | 1-(3-Chlorophenyl)Piperazine |
| 2.01 | 217.0848 | M+H | C_13_H_12_O_3_ | 2.39 | -5.07 | 94.85 | 3-Formyl-6-Isopropylchromone |
| 2.01 | 330.1661 | M+H | C_14_H_23_N_3_O_6_ | 2.44 | 0.42 | 83.65 | Valclavam |
| 2.03 | 161.0603 | M-H | C_10_H_10_O_2_ | 3.14 | -3.29 | 95.43 | Alpha-Methyl Cinnamic Acid |
| 2.03 | 281.1396 | M-H | C_15_H_22_O_5_ | 2.31 | 0.65 | 89.36 | Octyl Gallate |
| 2.05 | 591.1734 | M-H | C_28_H_32_O_14_ | 2.13 | 2.47 | 75.71 | Fortunellin/ Linarin |
| 2.10 | 235.1441 | M+H | C_13_H_18_N_2_O_2_ | 3.03 | 0.17 | 94.64 | Coumaroylputrescine |
| 2.11 | 200.0705 | M+H | C_12_H_9_NO_2_ | 2.98 | -0.61 | 93.04 | 3-Cyano-4,7-Dimethylcoumarin |

Continued

| **RT (min)** | **m/z** | **Adducts** | **Formula** | **Max fold change** | **Mass Error (ppm)** | **Isotope Similarity** | **Tentative Assignment** |
| --- | --- | --- | --- | --- | --- | --- | --- |
| 2.13 | 393.2102 | M+H | C_22_H_29_FO_5_ | 32.90 | 7.59 | 86.76 | Dexamethasone |
| 2.21 | 511.2352 | M+H | C_30_H_30_N_4_O_4_ | 2.40 | 2.43 | 84.03 | Deuteroporphyrin |
| 2.22 | 355.1049 | M-H | C_16_H_20_O_9_ | 2.02 | 4.15 | 92.30 | Gentiopicroside |
| 2.22 | 249.1126 | M+H | C_14_H_16_O_4_ | 2.07 | 1.98 | 85.04 | 6,7-Diethoxy-4-Methylcoumarin |
| 2.34 | 303.0505 | M+H | C_15_H_10_O_7_ | 9.51 | 1.75 | 92.61 | 3,7,3',4',5'-Pentahydroxyflavone |
| 2.34 | 465.1039 | M+H | C_21_H_20_O_12_ | 6.04 | 2.58 | 92.28 | Isoquercitin |
| 2.36 | 237.1602 | M+H | C_13_H_20_N_2_O_2_ | ∞ | 1.90 | 95.32 | Dropropizine |
| 2.40 | 156.1007 | M+H | C_8_H_13_NO_2_ | 2.67 | -7.80 | 83.41 | Scopoline |
| 2.43 | 212.0905 | M+H | C_10_H_13_NO_4_ | 2.78 | -5.67 | 63.55 | 6-Maleimidocaproic Acid |
| 2.47 | 373.2813 | M+H | C_23_H_36_N_2_O_2_ | 2.33 | -9.79 | 83.93 | Finasteride |
| 2.49 | 783.2541 | M+H | C_39_H_42_O_17_ | 3.75 | 5.86 | 62.80 | Spinosin 6'''-(E)-Ferulate |
| 2.50 | 215.0929 | M-H | C_10_H_16_O_5_ | 2.22 | 1.83 | 88.04 | 3-Oxo-1,8-Octanedicarboxylic Acid |
| 2.59 | 903.2548 | M+H | C_42_H_46_O_22_ | 6.04 | -0.62 | 85.84 | Isovitexin 2''-O-(6'''-(E)-P-Coumaroyl)Glucoside 4'-O-Glucoside |
| 2.62 | 287.0559 | M+H | C_15_H_10_O_6_ | 3.04 | 3.16 | 92.86 | Luteolin |
| 2.62 | 449.1119 | M+H | C_21_H_20_O_11_ | 2.15 | 9.11 | 92.43 | Luteolin-4'-O-Glucoside |
| 2.66 | 214.0877 | M+H | C_13_H_11_NO_2_ | 2.27 | 6.79 | 92.51 | 3-Cyano-4-Ethyl-6-Methylcoumarin |
| 2.73 | 175.0765 | M+H | C_11_H_10_O_2_ | 104000.00 | 6.53 | 88.37 | 3,6-Dimethylchromone |
| 2.73 | 402.2448 | M+H | C_27_H_31_NO_2_ | 2.11 | 5.20 | 78.29 | N-Didesmethylmifepristone (Ru 42848) |
| 2.75 | 209.0813 | M+H | C_11_H_12_O_4_ | 3.53 | 2.28 | 88.87 | 2,5-Dimethoxycinnamic Acid |
| 3.00 | 209.0460 | M-H | C_10_H_10_O_5_ | 2.1 | 1.92 | 97.25 | 5-Hydroxyferulate |
| 3.10 | 345.0612 | M+H | C_17_H_12_O_8_ | 2.55 | 2.07 | 86.86 | 3,5,3'-Trihydroxy-4'-Methoxy-6,7-Methylenedioxyflavone |
| 3.10 | 465.1034 | M+H | C_21_H_20_O_12_ | 2.52 | 1.42 | 89.90 | Isoetin 5'-Glucoside |
| 3.10 | 505.1005 | M-H | C_23_H_22_O_13_ | 2.36 | 3.47 | 85.89 | Quercetin 3-(6''-Acetylglucoside) |
| 3.10 | 609.1516 | M-H | C_27_H_30_O_16_ | 2.53 | 8.95 | 85.10 | Rutin |
| 3.10 | 175.0768 | M+H | C_11_H_10_O_2_ | 4.19 | 8.54 | 54.49 | 3,6-Dimethylchromone |
| 3.10 | 202.0874 | M-H | C_12_H_13_NO_2_ | 2.53 | 0.00 | 94.33 | 7-Dimethylamino-4-Methylcoumarin |
| 3.21 | 445.0785 | M-H | C_21_H_18_O_11_ | 2.76 | 1.99 | 86.32 | Baicalin |

Continued

| **RT (min)** | **m/z** | **Adducts** | **Formula** | **Max fold change** | **Mass Error (ppm)** | **Isotope Similarity** | **Tentative Assignment** |
| --- | --- | --- | --- | --- | --- | --- | --- |
| 3.21 | 596.1382 | M+Na | C_26_H_28_O_16_ | 1.48 | 0.84 | 97.03 | Myricetin 3-Xylosyl-(1->2)-Rhamnoside |
| 3.21 | 345.0612 | M+H | C_17_H_12_O_8_ | 2.00 | 1.93 | 89.94 | 3,5,3'-Trihydroxy-4'-Methoxy-6,7-Methylenedioxyflavone |
| 3.21 | 429.0828 | M+H | C_21_H_16_O_10_ | 4.21 | 2.72 | 86.22 | Theaflavic Acid |
| 3.21 | 465.1030 | M+H | C_21_H_20_O_12_ | 2.02 | 0.64 | 88.30 | Isoaffinetin |
| 3.21 | 597.3910 | M+H | C_40_H_52_O_4_ | 2.88 | -4.75 | 64.03 | Astaxanthin |
| 3.22 | 319.0460 | M+H | C_15_H_10_O_8_ | 4.17 | 3.54 | 91.02 | Myricetin |
| 3.30 | 465.1033 | M+H | C_21_H_20_O_12_ | 2.96 | 1.13 | 93.30 | Isoaffinetin |
| 3.36 | 257.1250 | M+Na | C_13_H_18_N_2_O_2_ | 2.21 | 1.92 | 95.21 | Coumaroylputrescine |
| 3.39 | 149.0603 | M+H | C_9_H_8_O_2_ | 2.16 | 4.08 | 99.16 | 3-Isochromanone |
| 3.49 | 881.3023 | M+H | C_41_H_52_O_21_ | 4.25 | -5.83 | 85.20 | Anhydroicaritin 3-(6'''-Acetylgalactosyl)(1->3)-Rhamnoside-7-Glucoside |
| 3.49 | 379.1165 | M-H | C_22_H_20_O_6_ | 2.5 | -5.86 | 92.51 | Robustic Acid |
| 3.53 | 319.0450 | M+H | C_15_H_10_O_8_ | 11.30 | 0.43 | 93.39 | Myricetin |
| 3.82 | 186.0553 | M+H | C_11_H_7_NO_2_ | 6.54 | 1.95 | 97.93 | 3-Cyano-6-Methylchromone |
| 3.82 | 195.0652 | M+H | C_10_H_10_O_4_ | 3.71 | 0.00 | 97.08 | Ferulic Acid |
| 3.83 | 448.1008 | M+Cl | C_21_H_20_O_11_ | 1.74 | 0.47 | 97.56 | Chrysanthemin |
| 3.84 | 353.0664 | M+H | C_19_H_12_O_7_ | 2.78 | 2.38 | 89.37 | Daphnoretin |
| 3.84 | 359.0774 | M+H | C_18_H_14_O_8_ | 2.94 | 3.44 | 90.03 | Dalpalatin |
| 3.84 | 395.0783 | M+H | C_21_H_14_O_8_ | 3.59 | 5.35 | 87.51 | Demethyltorosaflavone C |
| 3.84 | 449.1083 | M+H | C_21_H_20_O_11_ | 1.94 | 1.04 | 95.93 | 6-C-Galactosylisoscutellarein |
| 4.04 | 177.0548 | M+H | C_10_H_8_O_3_ | 2.60 | 1.26 | 98.93 | 8-Methoxycoumarin |
| 4.04 | 186.0550 | M+H | C_11_H_7_NO_2_ | 5.59 | 0.01 | 97.18 | 3-Cyano-6-Methylchromone |
| 4.04 | 195.0653 | M+H | C_10_H_10_O_4_ | 18.10 | 0.49 | 96.53 | Isoferulic Acid |
| 4.15 | 623.1621 | M+H | C_28_H_30_O_16_ | 2.74 | 2.29 | 79.22 | 6-Hydroxyluteolin 3'-Methyl Ether 7-[6''-(3-Hydroxy-3-Methylglutaryl)Glucoside] |
| 4.20 | 465.1046 | M+H | C_21_H_20_O_12_ | 2.73 | 4.04 | 83.87 | Hyperoside |
| 4.35 | 630.3331 | M+H | C_34_H_47_NO_10_ | 8.15 | 9.19 | 89.60 | Falaconitine |

Continued

| **RT (min)** | **m/z** | **Adducts** | **Formula** | **Max fold change** | **Mass Error (ppm)** | **Isotope Similarity** | **Tentative Assignment** |
| --- | --- | --- | --- | --- | --- | --- | --- |
| 4.42 | 341.0672 | M+H | C_18_H_12_O_7_ | 2.24 | 4.87 | 88.70 | 7-Methoxy-5,6:3',4'-Bis(Methylenedioxy)Flavone |
| 4.45 | 303.0505 | M+H | C_15_H_10_O_7_ | 3.34 | 1.89 | 92.95 | 5,7,3',4',5'-Pentahydroxyflavone |
| 4.66 | 463.1247 | M+H | C_22_H_22_O_11_ | 3.17 | 2.59 | 91.09 | Geraldol 4'-Glucoside |
| 4.68 | 223.0615 | M-H | C_11_H_12_O_5_ | 2.01 | 1.42 | 91.85 | Sinapic Acid |
| 4.69 | 755.2451 | M+H | C_34_H_42_O_19_ | 2.01 | 7.65 | 88.78 | 2'''-O-Rhamnosyl-2''-O-Glucosylcytisoside |
| 4.74 | 630.3326 | M+H | C_34_H_47_NO_10_ | 2.86 | 8.46 | 86.17 | Falaconitine |
| 4.75 | 495.1888 | M+H | C_24_H_30_O_11_ | 2.15 | 5.39 | 86.34 | Harpagoside |
| 4.80 | 163.0745 | M+H | C_10_H_10_O_2_ | 2.18 | -5.37 | 56.75 | 4-Methylcinnamic Acid |
| 5.39 | 471.1311 | M+H | C_24_H_22_O_10_ | 2.36 | 5.39 | 84.90 | Pongamoside |
| 5.40 | 461.1101 | M-H | C_22_H_22_O_11_ | 2.8 | 2.52 | 85.62 | Malvidin-3-O-Arabinoside |
| 5.74 | 435.1307 | M-H | C_21_H_24_O_10_ | 2.16 | 2.47 | 89.37 | Phloridzin |
| 6.28 | 755.2443 | M+H | C_34_H_42_O_19_ | 2.06 | 6.67 | 89.29 | Acacetin 7-O-[2'''-O-Rhamnosyl-2''-O-Glucosylglucoside |
| 6.29 | 291.1048 | M+H | C_12_H_18_O_8_ | 5.74 | -9.00 | 76.07 | Furaneol 4-Glucoside |
| 6.33 | 303.0504 | M+H | C_15_H_10_O_7_ | 2.02 | 1.62 | 94.06 | 3,6,2',4',5'-Pentahydroxyflavone |
| 6.34 | 709.2772 | M+H | C_34_H_44_O_16_ | 4.30 | 9.89 | 83.16 | 4'-Cinnamoylmussatioside |
| 6.45 | 177.0556 | M+H | C_10_H_8_O_3_ | 2.92 | 5.73 | 89.14 | 6-Hydroxy-4-Methylcoumarin |
| 6.52 | 207.0658 | M+H | C_11_H_10_O_4_ | 2.10 | 3.05 | 96.19 | 2-Hydroxy-8-Methylchromene-2-Carboxylate |
| 6.59 | 471.3469 | M+H | C_30_H_46_O_4_ | 2.46 | 0.02 | 83.99 | Glycyrrhetinic Acid |
| 6.59 | 489.3575 | M+H | C_30_H_48_O_5_ | 2.41 | 0.03 | 86.23 | Asiatic Acid |
| 6.61 | 628.1671 | M+H | C_27_H_31_O_17_ | 2.22 | 5.90 | 71.53 | Delphinidin 3-Glucosylglucoside |
| 6.63 | 473.3615 | M+H | C_30_H_48_O_4_ | 3.28 | -2.14 | 85.72 | Sumaresinolic Acid |
| 6.64 | 655.1678 | M+H | C_32_H_30_O_15_ | 3.27 | 3.13 | 85.49 | Malvidin 3-(6''-P-Caffeyglucoside) |
| 6.74 | 625.1568 | M+H | C_31_H_28_O_14_ | 2.10 | 2.66 | 87.64 | Peonidin 3-(6''-Caffeylgucoside) |
| 7.00 | 545.3843 | M+H | C_33_H_52_O_6_ | 15.20 | 1.11 | 86.42 | Ganoderic Acid |
| 7.00 | 927.4946 | M+H | C_47_H_74_O_18_ | 2.21 | -0.17 | 91.42 | Chikusetsusaponin |
| 7.17 | 593.3787 | M+H | C_37_H_52_O_6_ | 2.75 | -8.32 | 89.71 | Avenestergenin B2 |
| 7.36 | 646.3189 | M+H | C_34_H_47_NO_11_ | 2.48 | -5.08 | 66.94 | Aconitine |

Continued

| **RT (min)** | **m/z** | **Adducts** | **Formula** | **Max fold change** | **Mass Error (ppm)** | **Isotope Similarity** | **Tentative Assignment** |
| --- | --- | --- | --- | --- | --- | --- | --- |
| 7.38 | 177.0551 | M+H | C_10_H_8_O_3_ | 2.32 | 2.73 | 89.14 | 7-Methoxycoumarin |
| 7.63 | 397.3110 | M+H | C_27_H_40_O_2_ | 2.24 | 2.27 | 85.70 | Delta3,5-Deoxytigogenin |
| 7.63 | 415.3239 | M+H | C_27_H_42_O_3_ | 2.36 | 7.80 | 87.43 | Diosgenin |
| 7.66 | 503.3372 | M+H | C_30_H_46_O_6_ | 3.39 | 0.92 | 73.34 | Esculentic Acid (Phytolacca) |
| 7.70 | 287.0559 | M+H | C_15_H_10_O_6_ | ∞ | 3.00 | 91.81 | 3,6,3',4'-Tetrahydroxyflavone |
| 7.79 | 523.3693 | M+H | C_28_H_50_N_4O_3S | 2.23 | 3.26 | 84.36 | Oleic Acid-Biotin |
| 7.80 | 397.3104 | M+H | C_27_H_40_O_2_ | 2.21 | 0.79 | 86.56 | Alpha-Micropteroxanthin |
| 8.54 | 635.3955 | M+H | C_39_H_54_O_7_ | 2.91 | 2.03 | 83.57 | Trans-Coumaroylrotundic Acid |
| 8.73 | 503.3368 | M+H | C_30_H_46_O_6_ | 2.32 | 0.16 | 73.33 | Medicagenic Acid |
| 8.82 | 471.3470 | M+H | C_30_H_46_O_4_ | 2.03 | 0.27 | 76.35 | Beta.-Glycyrrhetinic Acid |
| 8.93 | 369.1193 | M+H | C_17_H_20_O_9_ | 2.27 | 3.58 | 89.69 | 5-O-Feruloylquinic Acid |
| 8.97 | 415.3213 | M+H | C_27_H_42_O_3_ | 3.89 | 1.54 | 79.97 | Diosgenin |
| 9.22 | 349.1989 | M+H | C_20_H_28_O_5_ | 2.02 | -5.94 | 87.42 | Cohumulone |
| 9.32 | 369.1198 | M+H | C_17_H_20_O_9_ | 3.41 | 4.92 | 90.71 | 4-O-Feruloylquinic Acid |
| 9.39 | 927.4598 | M+H | C_46_H_70_O_19_ | 2.42 | 1.53 | 76.29 | Spinacoside |
| 9.44 | 565.1557 | M+H | C_26_H_28_O_14_ | 3.14 | 0.85 | 82.19 | Vitexin 2”-O-xyloside |
| 9.59 | 487.3420 | M+H | C_30_H_46_O_5_ | 9.74 | 0.46 | 93.83 | Quillaic Acid |
| 9.68 | 487.3418 | M+H | C_30_H_46_O_5_ | 7.36 | -0.02 | 93.71 | Quillaic Acid |
| 9.90 | 927.4819 | M+H | C_50_H_70_O_16_ | 13.30 | 8.87 | 61.83 | Tragopogonsaponin B |
| 10.23 | 179.1073 | M+H | C_11_H_14_O_2_ | 2.63 | 3.56 | 95.30 | 5-Phenylvaleric Acid |
| 10.34 | 483.2921 | M+H | C_26_H_42_O_8_ | 2.65 | -6.59 | 85.40 | Fusicoccin |
| 10.48 | 369.1815 | M+H | C_21_H_24_N_2_O_4_ | 2.72 | 1.59 | 86.57 | Strictosidine Aglycone |
| 10.52 | 397.3080 | M+H | C_27_H_40_O_2_ | 2.30 | -5.26 | 53.79 | Tocotrienol |
| 11.15 | 397.3106 | M+H | C_27_H_40_O_2_ | 22.90 | 1.12 | 83.57 | Tocotrienol |
| 11.19 | 487.3359 | M+H | C_32_H_42_N_2_O_2_ | 4.21 | 8.26 | 84.93 | Docosahexaenoyl Serotonin |
| 11.35 | 413.3445 | M+H | C_28_H_44_O_2_ | 3.71 | 7.53 | 81.95 | Hydroxyvitamin D2 |

Continued

| **RT (min)** | **m/z** | **Adducts** | **Formula** | **Max fold change** | **Mass Error (ppm)** | **Isotope Similarity** | **Tentative Assignment** |
| --- | --- | --- | --- | --- | --- | --- | --- |
| 11.76 | 315.0493 | M-H | C_16_H_12_O_7_ | 3.97 | -5.46 | 85.02 | Quercetin 3'-Methyl Ether |
| 11.78 | 443.3663 | M+H | C_28_H_46_N_2_O_2_ | 2.67 | 6.94 | 72.74 | Stearoylserotonin |
| 12.33 | 589.4111 | M+H | C_35_H_56_O_7_ | 2.13 | 2.13 | 86.66 | Sanguisorbin |
| 12.41 | 489.3365 | M+H | C_33_H_44_O_3_ | 4.19 | 0.37 | 83.01 | 7',8'-Dihydro-8'-Hydroxycitraniaxanthin |
| 12.81 | 487.3416 | M+H | C_30_H_46_O_5_ | 4.61 | -0.43 | 88.04 | Quillaic Acid |
| 12.97 | 429.1155 | M-H | C_22_H_22_O_9_ | 3.05 | -8.46 | 88.54 | Ononin |
| 13.47 | 392.1329 | M+H | C_19_H_21_NO_8_ | 6.41 | -2.87 | 86.64 | Glycoperine |
| 13.79 | 287.0556 | M+H | C_15_H_10_O_6_ | 2.19 | 1.96 | 90.89 | 3,6,2',4'-Tetrahydroxyflavone |
| 14.16 | 503.3380 | M-H | C_30_H_48_O_6_ | 4.18 | 0.45 | 90.90 | Madecassic Acid |
| 14.18 | 487.3359 | M+H | C_32_H_42_N_2_O_2_ | 2.06 | 8.24 | 82.10 | Docosahexaenoyl Serotonin |
| 14.50 | 419.2438 | M+H | C_24_H_34_O_6_ | 2.41 | 2.39 | 89.29 | Phyllanthin |
| 14.93 | 443.2057 | M+H | C_25_H_30_O_7_ | 19.70 | -1.60 | 82.45 | Exiguaflavanone M |
| 15.45 | 219.1748 | M+H | C_15_H_22_O | 2.09 | 1.90 | 92.26 | Curlone |
| 18.60 | 489.3510 | M+Cl | C_30_H_46_O_3_ | 3.51 | 2.69 | 80.57 | Seocalcitol |
| 19.05 | 537.3950 | M+H | C_35_H_52_O_4_ | 4.31 | 2.15 | 84.85 | Hyperforin |
| 19.21 | 205.1236 | M+H | C_13_H_16_O_2_ | 6.50 | 6.43 | 86.44 | Butyl Cinnamate |
| 19.22 | 397.3428 | M+H | C_28_H_44_O | 1.99 | -9.43 | 90.06 | Ergocalciferol |
| 19.36 | 431.3138 | M+H | C_27_H_42_O_4_ | 1.99 | -4.09 | 90.83 | Hecogenin |

Peaks were putatively identified on the basis of accurate mass using MS^e^ fragmentation and isotope similarity, MS/MS and isotope distribution data which matched with NIST and METLIN metabolite databases using Progenesis QI 2.0.
